# Identification and treatment of residual and relapsed idiopathic clubfoot in 88 children

**DOI:** 10.1080/17453674.2018.1478570

**Published:** 2018-05-30

**Authors:** Jurre H Stouten, Arnold T Besselaar, M C (Marieke) Van Der Steen

**Affiliations:** 1 Department of Orthopaedic Surgery, Catharina Hospital Eindhoven, Eindhoven, The Netherlands;; 2 Orthopaedic Center Máxima, Máxima Medical Center, Eindhoven, The Netherlands

## Abstract

Background and purpose — The Ponseti treatment is successful in idiopathic clubfoot. However, approximately 11–48% of all clubfeet maintain residual deformities or relapse. Early treatment, which possibly reduces the necessity for additional surgery, requires early identification of these problematic clubfeet. We identify deformities of residual/relapsed clubfeet and the treatments applied to tackle these deformities in a large tertiary clubfoot treatment center.

Patients and methods — Retrospective chart review of patients who visited our clinic between 2012 and 2015 focused on demographics, deformities of the residual/relapsed clubfoot, and applied treatment. Residual deformities were defined as deformities that were never fully corrected and needed additional treatment. We defined relapse as any deformity of the clubfoot reoccurring, after initial successful treatment, with necessity for additional treatment.

Results — We identified 33 patients with residual and 55 patients with relapsed clubfeet. In both groups decreased dorsal flexion and adduction were the most often registered deformities. Furthermore, often equinus/decreased dorsiflexion, active supination, and varus occurred. In more than half, typical profiles of combined deformities were found. Relapses occurred at all stages of treatment and follow-up; half of the residual or relapsed clubfeet were identified before the end of the bracing period. In half of the patients, additional treatment consisted of the Ponseti treatment, one–quarter also required adaptation of the brace protocol, and one–quarter needed additional surgery. The Ponseti treatment was mainly reapplied if feet presented with relapses or residues until the age of 5.

Interpretation — Practitioners should especially be aware of equinus/decreased dorsiflexion, adduction, and active supination as a sign of a residual or relapsed clubfoot. Due to the heterogeneous profiles of these clubfeet, treatment strategy should be based on a step-by step approach including recasting, bracing, and if necessary surgical intervention.

The Ponseti treatment has shown to be a very successful treatment for idiopathic clubfoot (Ponseti et al. 1992, Morcuende et al. 2004). Unfortunately, approximately 11–48% of treated clubfeet show problems during follow up (Ponseti 2002, Morcuende et al. 2004, Hosseinzadeh et al. 2017). Some feet do not fully correct, while others have a tendency to relapse (Ponseti 2002). In the literature, the distinction between these problematic clubfeet is not always clear (Hosseinzadeh et al. 2017). In the current study we define residual deformities as deformities that underwent primary treatment but were never fully corrected and need additional treatment (Radler and Mindler 2015). A relapse is defined as any feature of the clubfoot reoccurring after initial successful treatment, which needs additional treatment (Laaveg and Ponseti 1980).

The pathology of residual or relapsed clubfeet is still unknown. It is clear that inappropriate bracing leads to relapses (Morcuende et al. 2004, Dobbs et al. 2004). But non-compliance with the bracing protocol does not explain all relapses. Any deformity of the initial clubfoot can be present in a residual or relapsed clubfoot. Furthermore, toe deformities, stiffness, or articular incongruence might be present (Uglow and Kurup 2010, Parsa et al. 2014).

The treatment of residual and relapsed clubfeet involves many challenges that were, in the past, frequently tackled by means of extensive surgical interventions (Radler and Mindler 2015). Nowadays, opinions have shifted and the additional treatment takes on a more reserved or nonoperative approach. A step-by-step approach is important and based on the original Ponseti treatment, involves repeated casting, a proper bracing period, and only if necessary surgical intervention (Dietz 2006, Jowett et al. 2011, Radler and Mindler 2015). The Ponseti treatment includes the possibility to expand the foot correction with tibialis anterior tendon transfer to redress the common problem of adduction and supination in residual and relapsed clubfeet (Parsa et al. 2014, Radler and Mindler 2015). Furthermore, treatment should be specific to the pathoanatomy of the deformity and functional needs of the patients should be taken into account during treatment planning (Radler and Mindler 2015). Applied to residual, relapsed, neglected, and complex clubfoot, the Ponseti treatment has shown positive results with respect to pain, functionality, and cosmesis (Dietz 2006, Lourenço and Morcuende 2007, Radler and Mindler 2015, Matar et al. 2016).

Early identification of residues and relapses allows for early treatment and therefore may diminish the necessity for major surgical interventions (Ponseti 2002, Dietz 2006). However, due to the variable manner in which a residual or relapsed clubfoot may occur, they may be difficult to identify at an early stage. Therefore, the aim of this study is to gain insight into the deformities of residual and relapsed clubfeet and the applied treatment at our clubfoot treatment center. Being aware which deformities occur most frequently and at what stage of the treatment aids in determining the optimal treatment and timing of this.

## Patients and methods

We performed a retrospective dossier study of clubfoot patients treated by one orthopedic surgeon specialized in clubfoot pathology (ATB) between 2012 and 2015. Potential study participants were identified by means of the Dutch diagnosis and treatment code (DBC) for clubfoot. Patients were included in the study if they had idiopathic clubfoot and underwent treatment for a residual or relapsed deformity of their clubfoot at our tertiary institute. Patients were excluded if they did not have idiopathic clubfoot but rather a syndromic, neurogenic, or positional clubfoot.

Data on demographics, clubfoot deformities, the primary treatment, and additional treatment were gathered from the electronic patient files. Known clubfoot deformities were recorded: adduction, equinus, varus, and cavus. Since decreased dorsiflexion and active supination are early signs of relapsing clubfeet and as such require treatment to prevent further problems, we documented these as well. Active supination is caused by suboptimal function of the tibialis anterior (TA) muscle. In residual or relapsed deformities, the shape of the foot results in relative medialization of the insertion of the TA. This leads to an over-supination in the early swing phase but also by landing on the lateral border of the foot. This mechanism can be studied during walking but also when sitting on a bench with the lower legs free. If the patient is asked to raise the foot, it can clearly be seen if the foot is dorsiflexed neutrally or with a supination component. Because equinus and decreased dorsiflexion are strictly related, and both mark a deformity in the sagittal plane of the ankle, we decided to combine the equinus and decreased dorsiflexion (EqDD) and treated them as a single entity.

The data on the deformities were gathered retrospectively and were cumulatively gathered per individual. Because of the retrospective character of this study, the moment at which specific deformities occurred could not be determined. Given these facts, the deformities in a single patient formed a profile of the deformities that occurred over time.

Primary treatment was defined as the initial treatment of any kind that was performed with the intention to fully correct the primary clubfoot. Treatment for residual or relapsed clubfoot that had been performed outside our own clinic was also recorded. Additional treatment comprised the treatment applied for a residual or relapsed clubfoot at our center. For the different treatment stages, we registered the date of the initial corrective casts, usage of braces, and any surgical treatment.

We composed 3 treatment groups that differ from each other in extensiveness of the additional treatment necessary to treat the residual or relapsed clubfoot. In the first group (extended Ponseti protocol) additional treatment consisted of additional treatment following the Ponseti protocol. This could entail a second casting phase, renewed Achilles tendon tenotomy (re-ATT), and/or a tibialis anterior tendon transfer (TATT) (Ponseti 2002). In the second group (brace adaptation), the treatment protocol of the Ponseti group was combined with adaptation of the bracing phase. In this group bracing was prolonged according to age or adjusted by the use of another type of brace. In those—often older—children who did not tolerate the standard foot abduction brace with a bar, an abduction dorsiflexion mechanism brace was used as an alternative. This brace consists of an alternative abduction, endorotation, dorsiflexion mechanism. It is constructed without a bar between both feet and therefore is usable unilaterally. The third group (additive surgery) entailed patients who received additional extra- and/or intra-articular surgical treatment that is not part of the aforementioned extended Ponseti protocol.

### Ethics, funding, and potential conflicts of interest

Ethics approval was obtained from the local medical ethical committee (niet-WMO 2016-23). No funding was obtained for this study. No conflicts of interest declared.

## Results

### Patient selection

**Figure 1.**
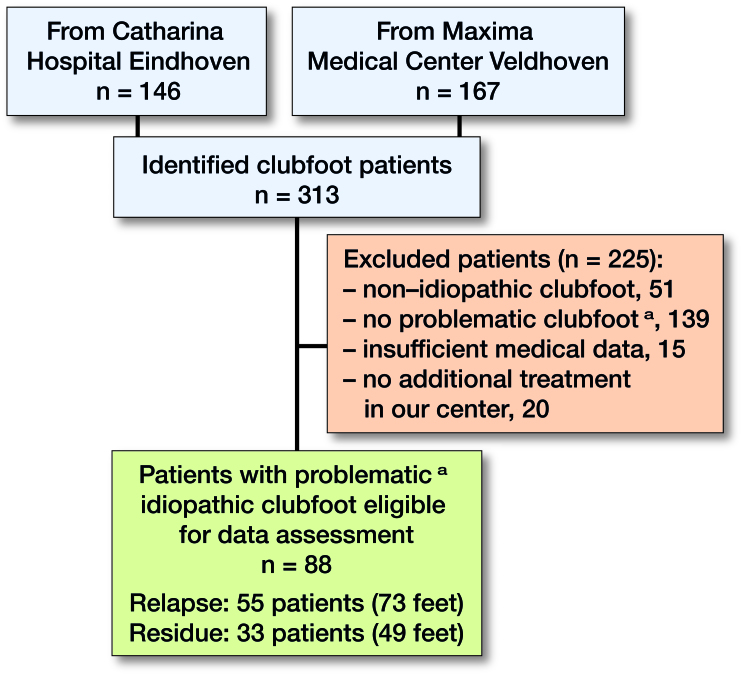
Flowchart of patient selection. **^a^**Residual and relapsed clubfeet.

Initially, we identified 416 patients by means of the diagnosis/treatment code (DBC) for clubfoot. First, 103 patients were excluded from the database because these consisted of mothers expecting a child with clubfoot who were counselled before delivery by the orthopedic surgeon or patients who had been labelled incorrectly as clubfoot patients, leaving 313 clubfoot patients. Ultimately, 88 patients with 122 residual or relapsed clubfeet were identified and included in the following analyses (Figure 1).

### Patient characteristics

The residual group comprised 33 patients (49 feet) and 55 patients (73 feet) had relapsed deformities. A substantial part (52/88) of the population was referred to our clinic from other centers. This explains the relatively high percentage of residual and relapsed feet in our population. As expected, the mean age at identification of the relapse was considerably higher than the age at identification of residual deformities (respectively 4.9 (0–15) years and 3.4 (0–16) years). The mean follow-up since identification of the residual and relapsed clubfoot was similar in both groups; in the residual group this was 3.8 (0.4–10) years, and in the relapse group 3.7 (0.3–21) years. (Table 1)

**Table 1.** Descriptive data on both groups for patients and feet

	Residual	Relapse
**Patients, n**	33	55
Age at identification in months		
mean (range)	41 (3–187)	59 (3–182)
Follow up since identification in months		
mean (range)	45 (5–120)	45 (3–250)
Male/female ratio (n)	4.5/1.0 (27/6)	2.0/1.0 (36/19)
Unilateral:bilateral ratio (n)	1.1/1.0 (17/16)	1.1/1.0 (29/26)
**Feet, n**	49	73
Pirani at start of primary treatment		
median (IQR) ^a^	5.8 (4.8–6.0)	5.5 (4.5–6.0)
Ponseti treatment used in primary treatment, n		
yes	26	49
no	19	16
missing	4	8
Number of casts used in Ponseti		
median (IQR) ^b^	8.0 (6.5–15.0)	5.0 (4.0–6.0)
Deformity, n		
equinus	20	12
decreased dorsiflexion	22	35
adduction	20	34
active supination	15	21
cavus	2	3
varus	13	13
Group of additional treatment, n		
Ponseti protocol	32	27
brace adaptation	8	23
additional surgery	9	23

**^a^**Data only available of 22/10 feet.

**^b^**Data only available of 37/17 feet.

The male-to-female ratio in the residual group was 4.5:1 and in the relapse group 2:1. Unilateral and bilateral clubfoot occurred frequently in the relapse group, as it did in the residual group (1.1:1.0). It should be noted that not all patients with bilateral clubfeet in the relapse group had a bilateral relapse. In the relapse group 8 patients with a bilateral clubfoot had a unilateral relapse.

Often Pirani scores and number of casts are used to give an idea of the primary severity of the clubfoot. Unfortunately, data on these two particular variables, especially of referred patients, were not complete (Table 1). Based on the available data, clubfeet in the residual group had a Pirani score at the start of the primary treatment of 5.8 with an interquartile range (IQR) of 4.8–6.0. For the relapse group the median Pirani score was 5.5 with an IQR of 4.5–6.0.

In the residual group the number of primarily treated patients according to the Ponseti method was 26 of 49 patients and a median of 8.0 casts were used (IQR =6.5–15) (Table 1). In the relapse group 49 of the 73 patients were treated with the Ponseti method as primary treatment and a median number of 5.0 casts was used to achieve initial correction (IQR =4.0–6.0).

### Deformities (Table 2)

Table 1 shows the occurrence of the different deformities. Portraying these deformities as single entities does shed some light on the deformities that appear in residual and relapsed clubfeet, but it does not grasp the full complexity of the clubfeet. In 26 of the residual clubfeet and 33 of the relapses, a profile with multiple deformities evolved over time. In total, 13 profiles with multiple deformities were identified in addition to 4 deformities occurring as a single entity (Table 2).

**Table 2.** Proportions of profiles of deformities in the relapse and residue group. Values are number of feet

Profile	Residual	Relapse
Single deformity	17	31
EqDD**^a^**	14	22
active supination	1	2
adduction	1	7
cavus	1	0
EqDD involved	16	20
EqDD + active supination	2	2
EqDD + adduction	5	4
EqDD + varus	0	1
EqDD + active supination + varus	1	0
EqDD + active supination + adduction	2	7
EqDD + adduction + cavus	0	1
EqDD + varus + adduction	4	3
EqDD + active supination + varus + adduction	2	2
Other combinations	10	13
active supination + adduction	3	4
active supination + varus	4	3
active supination + varus + adduction	0	1
varus + adduction	2	3
adduction + cavus	1	2

**^a^**EqDD = equinus/decreased dorsiflexion

EqDD occurred in 30 of the residual clubfeet and 42 of the relapsed clubfeet. In the majority of the profiles, EqDD played a role. Furthermore, EqDD is the most prevalent single deformity that occurs in residual and relapsed clubfoot. Adduction as a single entity, however, occurs in a rather large proportion in the relapse group as well. Adduction was present only once as a single entity in the residual group. Profiles in which active supination and adduction played a role were abundant as well (28 in the residue group and 41 in the relapse group). Varus was always combined with other deformities (Table 2).

**Figure 2.**
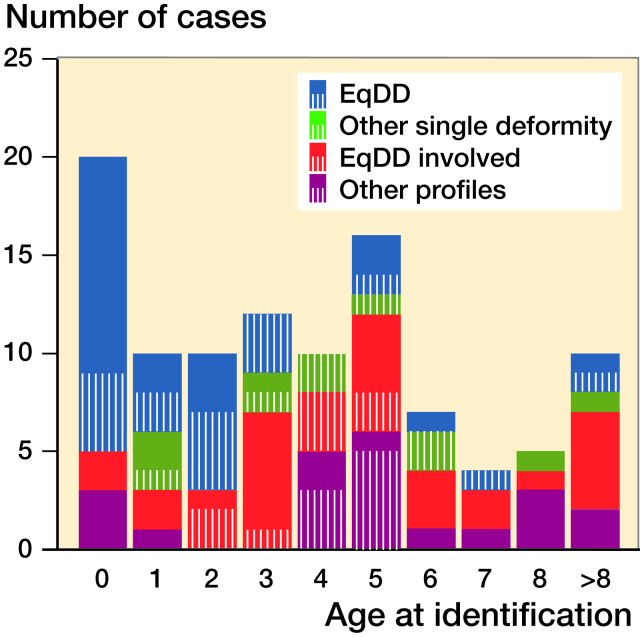
Age at identification of the residual and relapsed clubfoot compared to the group of profiles of deformities in the clubfoot. Blue bars show the feet with solitary equinus and/or decreased dorsiflexion and green bars are those with other solitary deformities. Red bars indicate feet with combined profiles that contain EqDD and purple bars show feet with other combined profiles. Plain tones show patients that were referred to our clinic.

As a means of examining the relation between profiles of deformities and the age at which the residual and relapsed clubfeet were initially identified, we plotted these variables against each other (Figure 2). As stated in the methods section, it should be noted that not all feet had sufficient data available to determine the age at identification (20/122 missing).

**Figure 3.**
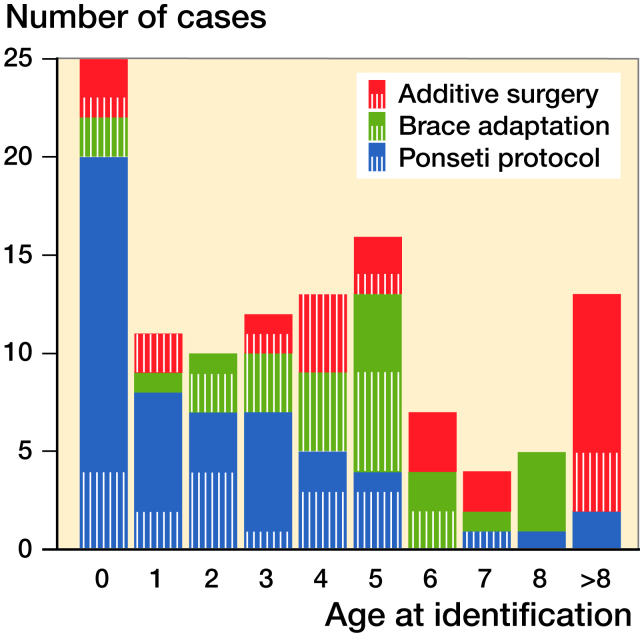
Age at identification of residual and relapsed clubfoot compared with their treatment group. Blue bars represent patients that had satisfying results with the Ponseti protocol. For patients displayed by green bars the Ponseti protocol was not sufficient and adaptation to a bracing protocol was needed to get good results. The red group contains those patients in which the previous 2 treatment options did not suffice and additive surgery was needed. Plain toned bars contain patients that were referred to our clinic.

An important proportion of the solitary EqDD deformity (27 of 36) occurs in the first year of life (Figure 3). When patients become older the amount of deformities occurring as a single entity diminishes. In our cohort 50% of the residual and relapsed clubfeet were identified before the end of the bracing period (before the age of 4). Furthermore, a peak is seen at the age of 5, 1 year after the end of the bracing period. After this peak, the amount of new residual and relapsed clubfeet decreases even further. Additionally, the proportion of clubfeet where single deformities play a role seems to decrease with age and these are seldom seen at the age of 6 and older. Combined deformities are detected more often as residual or relapsed deformities at a higher age. Figure 3 also distinguishes our own (striped) from referred patients (plain color). In the first two years of life patients are often referred with clubfeet that display EqDD. Referred patients often demonstrate a more complex profile as they are diagnosed later in life.

### Treatment groups

3 treatment groups were composed (see Methods section). The extended Ponseti protocol was sufficient in 32 out of 49 of the residue group and 27 out of the 73 clubfeet in the relapse group. An extra brace adaptation was sufficient in 8 of the residual clubfeet and in 9 of these feet additional surgery was needed (Table 1). In the relapse group 23 of the feet were treated with brace adaptation and 23 of the patients needed additional surgical interventions that were not a part of the Ponseti protocol.

The Ponseti protocol was mainly used when feet presented with residues or relapses until the age of 5 (Figure 2). Brace adaptations increase up to the age of 6, but by the age of 9 brace adaptation was not used in any of the cases. The additional surgery group was not influenced by age.

### Surgical interventions

In the 3 previously depicted treatment groups, ATT and TATT were considered part of the extended Ponseti protocol, whereas any other surgery was not part of the extended Ponseti protocol and was classified as additional surgery.

**Figure 4.**
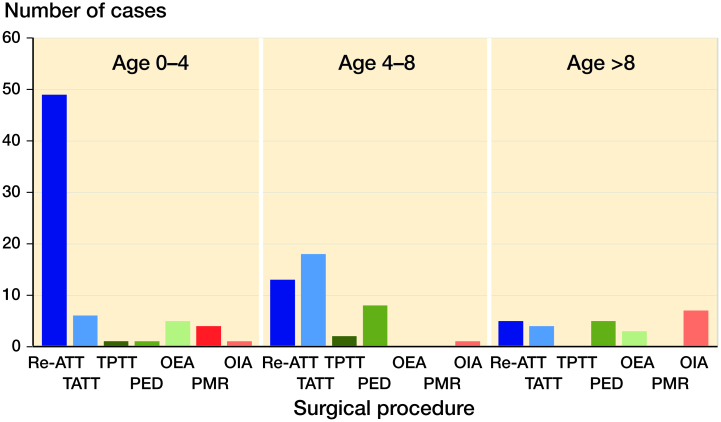
Age of patients at the moment of surgical intervention. Blue color marks the surgical treatments that are part of the Ponseti protocol. Green portrays extra-articular (EA) treatments that are not part of the Ponseti protocol. The red bars show the intra-articular (IA) treatments. Re-ATT: renewed Achilles tendon tenotomy, TATT: tibialis anterior tendon transfer, TPTT: tibialis posterior tendon transfer, PED: partial epiphysiodesis of the ventral distal tibia, OEA: other extra-articular surgery, PMR: posteromedial release, OIA: other intra-articular surgery.

Surgical treatment mostly consisted of extra articular procedures, of which re-ATT was applied 67 times out of 135 surgeries. Until the age of 4, re-ATT was almost exclusively the surgical treatment of choice. After that, TATT was used more often as well as other extra articular procedures (Figure 4).

The additional surgeries which are not part of the Ponseti protocol (see Figure 4, in green and red) were mostly preserved for older children in whom other treatment did not prove to be effective. We subdivided these additional surgeries into extra-articular treatments (green) and intra-articular treatments (red). The partial epiphisiodesis (PED) of the anterior segment of the distal end of the tibia was the most predominantly used extra-articular treatment that was not part of the Ponseti protocol. Intra-articular surgeries (see Figure 4, in red) were used in older children as well and consisted of posteromedial releases, closing wedge osteotomies, a triple arthrodesis, a fascia plantaris release, and the use of external fixators (in medial column lengthening and metatarsal osteotomies).

## Discussion

We describe a cohort of residual and relapsed clubfeet treated at a tertiary clubfoot treatment center. By identifying the profiles of deformities occurring in these problematic clubfeet and describing the treatment performed we depict the strength of the Ponseti treatment used in these patients but also show the necessity for additional surgical interventions in some patients.

Our population showed high resemblance to the normal clubfoot population in terms of affected foot and sex (Werler et al. 2013). The majority of the included patients had already initially been treated with the Ponseti method, nowadays the preferred initial treatment for idiopathic clubfeet in the Netherlands (Besselaar et al. 2017). The residual group showed a high number of casts used (median 8 casts), while the initial Pirani score was not higher. This suggests that these clubfeet already showed difficulties during the initial correction. Zhao et al. (2016) also showed that difficulty in correcting the initial deformity was predictive for a relapse. Furthermore, Ponseti et al. (2006) noted a specific group of complex clubfeet that are difficult to treat and require a modified Ponseti treatment.

All known clubfoot deformities could be present in residual and relapsed clubfeet. As was pointed out by Ponseti in 2002, equinus/decreased dorsiflexion is the most frequent reoccurring deformity, as we found as well. We also found many different profiles of deformities, though deformities in children younger than 4 years old often occurred solitarily.

The majority of relapses occur in the first 2 to 3 years of life and rarely after the age of 5 (Dietz 2006, Ponseti et al. 2006). However, we also saw an increase in cases at the end of the bracing period. This difference might be related to ours being a tertiary clubfoot treatment center. As a consequence, patients may be older at presentation than they would be in other centers. However, the peak of residuals and relapses around the age of 5 might also point toward the important role of the foot adduction brace in preventing relapses. It should, however, be noted that difficulties during bracing often are a sign of a residual or relapsed foot causing a fitting problem as a result of decreased dorsiflexion (Dietz 2006). In our study, however, in children over the age of 5 relapses often occurred in more complex deformity profiles.

Deformities, age, and treatment are all associated with each other, as age and deformities determine the suitable treatment. Treatment using the Ponseti protocol is particularly prevalent in the first 4 years of life, while brace adaptations and additional surgery are commoner in older children; we found the same in our cohort. This is in line with the step-by-step approach described by Radler and Mindler (2015) and Jowett et al. (2011). Early identification seems to be essential in preventing the need for additional surgical interventions, which have been associated with less positive outcomes in pain, functionality, and cosmesis (Radler and Mindler 2015). In our retrospective cohort no objective scores on pain, functionality, and cosmesis were available.

Of course, the retrospective nature of our study also imposes several other limitations. The most important of these are incomplete data and difficulty distinguishing between residual and relapsed clubfeet, especially in referred cases. We defined a relapse in line with the Iowa group. Radler and Mindler (2015) suggest that the differentiation has minimal effect on further treatment. Perhaps a classification of residual and relapsed clubfeet based on severity might be more useful. However, it is difficult to compare the severity of the different profiles of deformities. Bhaskar and Patni (2013) identified 5 relapse patterns based on the involvement of dorsiflexion, adduction, or supination and whether the deformity was either dynamic or fixed. As we found many profiles of deformities, regularly also including cavus and varus, we felt that the Bhaskar classification was not capable of embracing the complexity of both residual and relapsed clubfeet. It should be noted that as we are a tertiary center, we generally treat the more severe cases and therefore the profiles of deformities might be more complex than in a standard clubfoot population.

In summary, our study showed that relapses occur at all stages of treatment and follow-up. All deformities of the initial clubfeet can (re)occur in residual and relapsed clubfeet and often a combination of deformities is seen. Practitioners should especially be aware of EqDD, adduction, and active supination as signs of a relapse. Due to the heterogeneous nature of residual and relapsed clubfeet, the treatment strategy should be based on a step-by-step approach including recasting, bracing, and if necessary surgical intervention. In the majority of our cases, especially if identified in an early stage, treatment according to Ponseti was sufficient to treat the residual and relapsed clubfeet. Identifying residues or relapses at an early stage could prevent the need for additional surgery.

JHS: Data collection, analysis and, interpretation. Drafting the article. MCS: Conception or design of the work. Data interpretation. Drafting the article. ATB: Conception or design of the work. Data interpretation. Critical revision of the article.

*Acta* thanks Klaus Dieter Parsch and other anonymous reviewers for help with peer review of this study.

## References

[CIT0001] Besselaar A T, Sakkers R J B, Schuppers H A, Witbreuk M M E H, Zeegers E V C M, Visser J D, Boekestijn R A, Margés S D, Van der Steen M C M, Burger K N J. Guideline on the diagnosis and treatment of primary idiopathic clubfoot. Acta Orthop 2017; 88(3): 305–9. 28266239 10.1080/17453674.2017.1294416 PMC5434600

[CIT0002] Bhaskar A, Patni P. Classification of relapse pattern in clubfoot treated with Ponseti technique. Indian J Orthop 2013; 47(4): 370–6. 23960281 10.4103/0019-5413.114921 PMC3745691

[CIT0003] Dietz F R. Treatment of a recurrent clubfoot deformity after initial correction with the Ponseti technique. Instr Course Lect 2006; 55: 625–9. 16958495

[CIT0004] Dobbs M B, Rudzki J R, Purcell D B, Walton T, Porter K R, Gurnett C A. Factors predictive of outcome after use of the Ponseti method for the treatment of idiopathic clubfeet. J Bone Joint Surg Am 2004; 86-A(1): 22–7. 14711941 10.2106/00004623-200401000-00005

[CIT0005] Hosseinzadeh P, Kiebzak G M, Dolan L, Zionts L E, Morcuende J. Management of clubfoot relapses with the Ponseti method: results of a survey of the POSNA members. J Pediatr Orthop 2017; Feb 7. doi: 10.1097/BPO.0000000000000953. [Epub ahead of print] 28178093

[CIT0006] Jowett C R, Morcuende J A, Ramachandran M. Management of congenital talipes equinovarus using the Ponseti method: a systematic review. J Bone Joint Surg Br 2011; 93(9): 1160–4. 21911524 10.1302/0301-620X.93B9.26947

[CIT0007] Laaveg S J, Ponseti I V. Long-term results of treatment of congenital club foot. J Bone Joint Surg Am 1980; 62(1): 23–31. 7351412

[CIT0008] Lourenço A F, Morcuende J A. Correction of neglected idiopathic club foot by the Ponseti method. Bone Joint J 2007; 89-B(3): 378–81. 10.1302/0301-620X.89B3.18313 17356154

[CIT0009] Matar H E, Bierne P, Bruce C E, Garg N K. Treatment of complex idiopathic clubfoot using the modified Ponseti method: up to 11 years follow-up. J Pediatr Orthop B 2016; 26(2): 137–42. 10.1097/BPB.0000000000000321 27104942

[CIT0010] Morcuende J A, Dolan L A, Dietz F R, Ponseti I V. Radical reduction in the rate of extensive corrective surgery for clubfoot using the Ponseti method. Pediatrics 2004; 113(2): 376–80. 14754952 10.1542/peds.113.2.376

[CIT0011] Parsa A, Moghadam M H, Jamshidi M H . Relapsing and residual clubfoot deformities after the application of the Ponseti method: a contemporary review. Arch Bone Jt Surg 2014; 2(1): 7–10. 25207306 PMC4151443

[CIT0012] Ponseti I. Current concept review: treatment of congenital club foot. J Bone Joint Surg 1992; 74A(3): 448. 1548277

[CIT0013] Ponseti I V. Relapsing clubfoot: causes, prevention and treatment. Iowa Orthop J 2002; 22: 55–6. 12180612 PMC1888384

[CIT0014] Ponseti I V, Zhivkov M, Davis N, Sinclair M, Dobbs M B, Morcuende J A. Treatment of the complex idiopathic clubfoot. Clin Orthop Relat Res 2006; 451: 171–6. 16788408 10.1097/01.blo.0000224062.39990.48

[CIT0015] Radler C, Mindler G T. Treatment of severe recurrent clubfoot. Foot Ankle Clin 2015; 20(4): 563–86. 26589079 10.1016/j.fcl.2015.07.002

[CIT0016] Uglow M G, Kurup H V. Residual clubfoot in children. Foot Ankle Clin 2010; 15(2): 245–64. 20534354 10.1016/j.fcl.2010.01.003

[CIT0017] Werler M M, Yazdy M M, Mitchell A A, Meyer R E, Druschel C M, Anderka M, et al Descriptive epidemiology of idiopathic clubfoot. Am J Med Genet A 2013; 161A(7): 1569–78. 23686911 10.1002/ajmg.a.35955 PMC3689855

[CIT0018] Zhao D, Li H, Kuo K N, Yang X, Wu Z, Liu J, Zhu J. Prognosticating factors of relapse in clubfoot management by Ponseti method. Pediatr Orthop B 2016; Sep 22. Epub 10.1097/BPO.0000000000000870 27662384

